# Chaperone-Mediated Autophagy Markers LAMP2A and HSC70 Are Independent Adverse Prognostic Markers in Primary Resected Squamous Cell Carcinomas of the Lung

**DOI:** 10.1155/2020/8506572

**Published:** 2020-09-21

**Authors:** Tereza Losmanová, Félice A. Janser, Magali Humbert, Igor Tokarchuk, Anna M. Schläfli, Christina Neppl, Ralph A. Schmid, Mario P. Tschan, Rupert Langer, Sabina Berezowska

**Affiliations:** ^1^Institute of Pathology, University of Bern, Bern 3008, Switzerland; ^2^Graduate School for Cellular and Biomedical Sciences, Bern 3012, Switzerland; ^3^Division of General Thoracic Surgery, Inselspital University Hospital Bern, Bern 3010, Switzerland; ^4^Department of Biomedical Research (DBMR), University of Bern, Bern 3008, Switzerland; ^5^Institute of Pathology and Molecular Pathology, Kepler University Hospital, Johannes Kepler University Linz, 4021 Linz, 4040 Linz, Austria; ^6^Institut de Pathologie, Centre Hospitalier Universitaire Vaudois et Université de Lausanne, Lausanne 1011, Switzerland

## Abstract

LAMP2A and HSC70 are crucial players in chaperone-mediated autophagy (CMA), a targeted, lysosome-dependent protein degradation pathway. Elevated LAMP2A levels, indicative of increased CMA activity, are observed in several malignancies, and CMA downregulation may be exploited therapeutically. We evaluated the impact of LAMP2A and HSC70 in pulmonary squamous cell carcinomas (pSQCC). Antibodies were validated by knockdown and overexpression experiments using three different cell lines. Expression levels in tissue were analyzed by immunohistochemistry in a cohort of 336 consecutive pSQCC using tissue microarrays. There was no significant correlation between the two markers among each other and no association with pathological parameters (TNM categories, grading). However, both high LAMP2A and HSC70 expression were associated with worse outcome, including overall survival (OS; *p* = 0.012 and *p* = 0.001) and disease free survival (DFS; *p* = 0.049 and *p* = 0.036). In multivariate analysis, both markers and a combination of them were independent adverse prognostic factors for OS (LAMP2Ahigh: HR = 2.059; *p* < 0.001; HSC70high: HR = 1.987; *p* < 0.001; LAMP2Ahigh/HSC70high: HR = 1.529; *p* < 0.001) and DFS (LAMP2Ahigh: HR = 1.709; *p* = 0.004; HSC70high: HR = 1.484; *p* = 0.027; LAMP2Ahigh/HSC70high: HR = 1.342, *p* < 0.001). The negative prognostic impact of high LAMP2A and HSC70 and their variable expression in pSQCC may justify the use of these proteins as potential biomarkers for future CMA-inhibiting therapies.

## 1. Introduction

**Figure 1:**
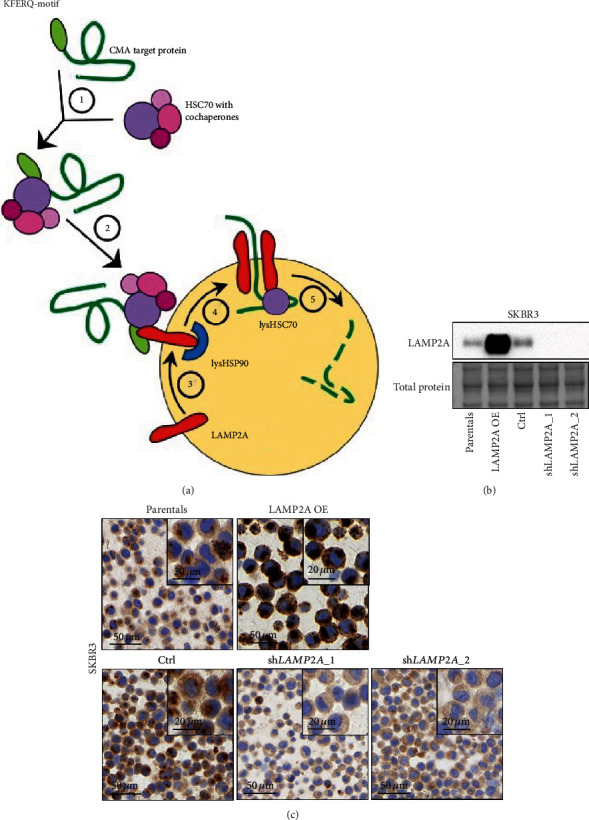
CMA pathway and validation of LAMP2A immunohistochemical staining. (a) Overview of chaperone-mediated autophagy (CMA). ① Recognition and binding of HSC70 to the KFERQ-motif of the target protein. ② Translocation of the complex to the lysosome. ③ Binding of the target protein to LAMP2A at the lysosomal membrane. ④ Formation of a multimeric LAMP2A complex. ⑤ Translocation and degradation of the target protein. (b, c) Specificity of LAMP2A immunohistochemistry. SKBR3 cells were transduced with lentiviral vectors containing a LAMP2A cDNA (OE) construct, an empty vector control, or shRNAs targeting LAMP2A mRNA (shLAMP2A_1-2). After selection, cells were subjected to LAMP2A western blot analysis (b) and immunohistochemistry (c).

Autophagy describes different lysosomal degradation pathways targeting damaged cytosolic proteins and organelles. Deregulation of autophagy pathways is involved in many physiological and pathophysiological mechanisms such as cell aging, neurodegenerative disorders, lysosomal storage diseases, and cancer [1]. However, the role of autophagy in tumorigenesis and its prognostic impact is complex and not fully understood. The term autophagy encompasses three main pathways (macroautophagy, microautophagy, and chaperone-mediated autophagy (CMA)) that differ in how the targeted cytosolic content reaches the lysosome for degradation [2]. In contrast to the highly conserved process of macroautophagy, CMA is only observed in mammalian cells. It is involved in the quality control of proteins by selectively degrading altered or damaged proteins. The process is induced upon different stresses as for instance hypoxia, and it is maximally activated upon prolonged cell starvation [3, 4]. Briefly, CMA specific client proteins bear a specific pentapeptide stretch, the KFERQ-like motif (Lys-Phe-Glu-Arg-Gln) [5], which is recognized in the cytosol by the heat shock cognate protein of 70 kDa (HSC70, also known as HSPA8) within a cytosolic chaperone complex. Then, the client protein is shuttled to the lysosome where it is unfolded and translocated into the lysosome through a multimeric complex of lysosome-associated membrane protein 2A (LAMP2A) (Figure 1(a)) [6]. The binding of the CMA target protein to LAMP2A monomer initiates a multimerization process involving several LAMP2A proteins. They form a translocation complex through which the unfolded target protein is translocated for degradation into the lysosomal lumen (Figure 1(a)) [7, 8]. Since the expression and degradation of LAMP2A is tightly regulated, this protein is considered the rate-limiting factor of the CMA process.

HSC70 is a heat shock protein (HSPs). HSPs are a large group of chaperones, which are induced upon different stresses. A subclass is formed by the HSP70 family, which includes at least 13 proteins including HSC70. This chaperone is present at the cellular membrane, extracellular exosomes, the nucleus, and the cytosol [9]. Its main function is protein quality control, where it acts as a folding catalyst or targets misfolded proteins for degradation [10, 11].

LAMP2A on the other hand is an alternative splice variant of the protein encoded by LAMP2. LAMP2 is a transmembrane glycoprotein in the lysosomal membrane with three splice variants (LAMP2A, B, and C). The three isoforms share some functions such as antigen presentation, cholesterol trafficking, lysosome biogenesis, and phagocytosis while some are specific to each isoform [12]. For instance, LAMP2A is the unique LAMP2 essential for CMA.

As observed in several human cancer cell lines and in primary tumor samples, CMA seems activated in different cancer types, evidenced by markedly increased LAMP2A levels [13, 14]. In vitro, inhibition of CMA leads to decreased tumor cell survival, and in mouse cancer xenograft models, CMA inhibition results in reduced metastases and tumor shrinkage [13, 15]. However, this rather tumor supportive effect of CMA is not fully understood, and it is important to emphasize that under physiological conditions, CMA is rather tumor suppressive [16]. Data on the expression of CMA-related proteins in human cancers and their potential impact on tumor aggressiveness or response to anticancer treatment are still scarce.

Non-small cell lung cancer (NSCLC) is the leading cause of cancer death in all European countries and worldwide [17]. Among NSCLC, pulmonary squamous cell carcinoma (pSQCC) is the second most common histological subtype. It is also a subtype with a strong association to cigarette smoking [18]. The influence of carcinogens in the cigarette smoke results in a high rate of genetic and epigenetic alterations in each tumor [19]. In contrast to adenocarcinomas, pSQCC usually lack any of the main therapeutic targets like mutations in EGFR or ALK fusions [20, 21]. In recent years, new therapeutic options using immunotherapy were developed, but the benefit for most of the patients with pSQCC is still limited, and there is a need to explore alternative approaches [22, 23].

In our study, we aimed at determining the expression patterns and the prognostic relevance of LAMP2A and HSC70, the two key players of CMA, in pulmonary SQCC.

## 2. Materials and Methods

### 2.1. Cell Lines and Culture Conditions

The human acute promyelocytic leukemia (APL) cell line, NB4, was obtained from the German Collection of Microorganisms and Cell Cultures GmbH (DSMZ, Braunschweig, Germany), and the SKBR3 breast cancer cells were a kind gift of Professor E. Garattini (Mario Negri Institute for Pharmacological Research, Milano, Italy). NB4 cells were maintained in RPMI-1640 with 10% fetal calf serum (FCS), 50 U/mL penicillin, and 50 *μ*g/mL streptomycin, and the SKBR3 cells were cultured in DMEM/F12, 5% FCS, 50 U/mL penicillin, and 50 *μ*g/mL streptomycin. Cells were kept at 5% CO2-95% air humidified atmosphere at 37°C. The human embryonic kidney (HEK) 293 cells expressing SV40-T-antigen (293 T) were a kind gift of Professor B. E. Torbett (Scripps Research, La Jolla, CA). 293 T cells were maintained in DMEM (Sigma-Aldrich, St. Louis, MO, USA), supplemented with 5% FBS, 1% penicillin/streptomycin, and 1% Hepes (Sigma-Aldrich), and kept in 7.5% CO2-95% air humidified atmosphere at 37°C.

### 2.2. Cell Lysate Preparation and Western Blotting

Whole cell extracts were prepared using UREA lysis buffer, and 30-60 *μ*g total protein was loaded on a 12% denaturing polyacrylamide self-cast gel (Biorad). Blots were incubated with the primary antibodies in TBS 0.05% Tween-20/5% milk overnight at 4°C (anti-HSC70, Thermofisher MA3-014; anti-LAMP2A, Abcam 125068), incubated with HRP-coupled secondary goat anti-rabbit and goat anti-mouse antibody (cell signaling) at 1 : 5–10,000 for 1 h at room temperature.

### 2.3. Transient Transfection and Lentiviral Vectors

**Figure 2:**
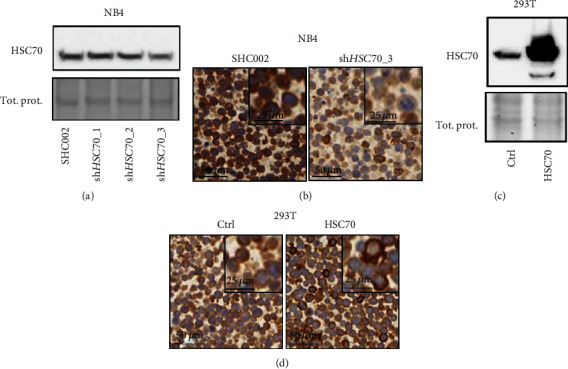
Validation of HSC70 immunohistochemical staining. (a, b) HSC70 knockdown in NB4 APL cells. (a) HSC70 knockdown efficiency of three independent shRNAs (shHSC70_1-3) was determined by western blotting and comparison to scramble shRNA transduced control cells (SHC002). (b) The most efficient HSC70 knockdown (shHSC70_3) was selected and subjected to immunohistochemistry. (c, d) 293 T cells were transiently transfected with an empty vector (ctrl) and HSC70 expression plasmid. (c) HSC70 expression was validated by western blotting. (d) 293 T cells were subjected to HSC70 immunohistochemistry.

HEK 293 T cells were transiently transfected with plasmid pLX307 encoding for HSC70 (HSPA8) using the calcium phosphate method [24]. pLKO.1-puro lentiviral vectors expressing shRNAs targeting HSC70 (shHSC70_1: NM_006597.3-976s1c1, shHSC70_2: NM_006597.3-335s21c1, shHSC70_3: NM_006597.3-2040s21c1) were purchased from the Sigma-Aldrich. These vectors contain a puromycin antibiotic resistance gene for selection of transduced mammalian cells. Sequences of shRNAs to target LAMP2A were (1) shRNA: CTGCAACCTGATTGATTA and (2) shRNA: GGCAGGAGTACTTATTCTAGT. These shRNA sequences were cloned into a U6-EF1a-IRES-hygro lentiviral vector backbone. Lentivirus production and transduction were done as described [25, 26]. Transduced NB4 and SKBR3 cell populations were selected with 1.5 *μ*g/mL puromycin for 4 days, and knockdown efficiency was assessed by western blot analysis (Figure 2).

### 2.4. Patient Cohort

In this single center, retrospective study, we investigated a consecutive cohort of patients with primary resected pSQCC, diagnosed at the Institute of Pathology, University of Bern, between 01/2000 and 12/2013. The study was performed according to the REMARK-guidelines and was approved by the Cantonal Ethics Commission of the Canton of Bern (KEK 200/14), which waived the requirement for written informed consent. As previously described, 402 patients met the inclusion criteria of the diagnosis pSQCC according to pathological records [27]. Finally, we included only tumors with confirmed squamous differentiation according to retrospectively performed immunohistochemical staining for p40 and TTF-1, according to current guidelines. Additionally, we excluded patients with previous or concomitant diagnosis of primary SQCC of other organ systems in order to reliably exclude metastatic lung disease and patients whose tumors were resected after neoadjuvant therapy according to reevaluation of clinical files. Tumors were restaged according to the 8th edition of the UICC TNM-classification [28, 29]. Tumor grading was reevaluated in all cases as previously described [30]. In short, grading was performed according to the cancer grading manual that evaluates the microscopic extension of keratinization, similar to the grading of SQCC of other anatomical regions. Grade 1 was assigned to tumors with prominent keratinization and/or prominent intercellular bridges. Grade 2 was assigned to tumors with scattered foci of keratinization, less prominent intercellular bridges, smaller tumor cells, or central comedo-like necrosis. Grade 3 tumors showed only rare or missing intercellular bridges, no keratin pearls formation, sheet-like growth, or single cell infiltration. Grade 1 and 2 corresponded to the WHO classification category of keratinizing carcinomas, and Grade 3 depicted nonkeratinizing carcinomas [31].

Finally, 354 primary resected pSQCC were available for immunohistochemical analysis. Out of these cases, LAMP2A and HSC70 could be evaluated in 336 tumors. For the remaining cases, there was no sufficient tumor material in the TMA cores, or the immunoreactivity of the tissue was insufficient due to technical error. Detailed clinicopathological characteristics are provided in Table 1. Adjuvant chemotherapy or radiotherapy was administered in 116 patients (35%).

### 2.5. Next-Generation Tissue Microarray

Immunohistochemical staining was applied on a next generation tissue microarray (ngTMA) constructed as previously described, with digital annotation of scanned slides and automatic transfer of the punches [32]. Two separate ngTMAs with a total of four punches per tumor (diameter = 0.6 mm) randomly taken from different tumor regions were used.

### 2.6. Immunohistochemical Staining and Scoring

Immunohistochemical staining for LAMP2A and HSC70 was done on 4 *μ*m sections using an automated immunostainer Leica Bond RX (Leica Biosystems, Heerbrugg, Switzerland) with the following conditions (dilution, antigen retrieval): LAMP2A (Novus Biologicals, Zug, Switzerland, rabbit polyclonal, #NB600-1384): 1 : 500, tris buffer, 95°C, and 30 min; and HSC70 (LabForce mbl, Nunningen, Switzerland, rabbit polyclonal, #PM0045): 1 : 10,000, citrate buffer, 100°C, and 30 min. For visualization, the Bond Polymer Refine Detection kit (Leica Biosystems, Muttenz, Switzerland, DS9800) was used according to the instructions of the manufacturer.

Scoring of LAMP2A and HSC70 was performed by a pathologist (TL) on a Zeiss Axioscope microscope at 10x objective magnification for each TMA core separately. We assessed the staining intensity in tumor cells ranging from 0 (negative), 1 (weak), 2 (medium) to 3 (strong). The percentage of stained tumor cells was determined using the following increments: 0 ≤ 5%, 1 = 6‐25%, 2 = 26‐50%, 3 = 51‐75%, and 4 = 76‐100%. Finally, the immunoreactivity score (IRS) was calculated by multiplication of the scores for intensity with the scores of the percentages of positive tumor cells.

**Figure 3:**
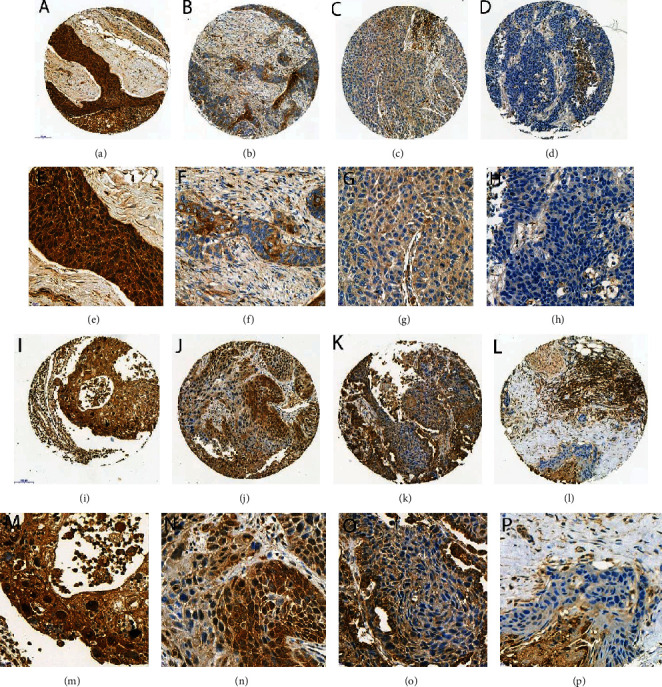
Examples of immunohistochemical staining: (a–h) LAMP2A ((a, e) IRS 3x4 = 12; (b, f) IRS 3x2 = 6; (c, g) IRS 1x4 = 4; (d, h) IRS 0x0 = 0); (i–p) HSC70 ((i, m) IRS 3x4 = 12; (j, n) IRS 3x3 = 9; (k, o) IRS 1x4 = 4; (l, p) IRS 0x0 = 0). Objective magnification: (a–d) 13x, (e–h) 40x, (i–l) 13x, and (m–p) 40x.

The staining was cytoplasmatic for LAMP2A and HSC70. Some cases showed both cytoplasmatic and nuclear HSC70 staining. The necrotic areas were strongly positive for both markers and discarded from the evaluation. The examples of staining are shown in Figure 3.

The individual IRS was used to assess intratumoral heterogeneity. For the final determination of the marker expression level in the tumor, the sum of the IRS over all cores divided by the number of cores was calculated for each tumor. The IRS sum score was used for the correlation of the marker expression with pathological parameters. For survival analysis, the cohort was first divided into quartiles. The best prognostic differentiation was observed by stratification of the results in low expression (lower three quartiles) and high expression (fourth quartile).

### 2.7. Statistical Analysis

IBM SPPS Statistics 26 (IBM Corporation, Armonk, USA) was used for statistical analyses. For group comparisons, crosstabs, X2 tests, and Fisher's exact tests were used. Survival analysis (overall survival and disease free survival) was calculated from the day of surgery. For univariate survival analysis, the Kaplan-Meier curves and log-rank tests were used. For multivariate survival analysis, the Cox regression analysis was used. *p* values of <0.05 were considered as significant for all tests.

## 3. Results

### 3.1. Validation of LAMP2A And HSC70 Antibodies for Immunohistochemistry

First, we validated the specificity of the antibodies LAMP2A and HSC70 for immunohistochemical staining. We generated a series of LAMP2A and HSC70 knockdown and overexpression cell lines. For this, we used lentiviral vectors to express LAMP2A cDNA as well as two independent shRNAs targeting LAMP2A in SKBR3 breast cancer cells. We confirmed ectopic expression and knockdown efficiency of LAMP2A by western blot analysis (Figure 1(b)). We detected a marked overexpression of LAMP2A compared to parental SKBR3 cells in cells expressing the exogenous LAMP2A cDNA. In addition, expression of both shRNAs targeting LAMP2A resulted in an efficient depletion of LAMP2A in SKBR3 cells compared to control transduced cells. Next, FFPE cell pellets were subjected to LAMP2A immunohistochemical staining. Consistent with the western blot data, immunohistochemical analysis revealed increased or depleted LAMP2A expression in LAMP2A cDNA and shLAMP2A transduced cells, respectively (Figure 1(c)). Of note, in agreement with the lysosomal localization of LAMP2A during CMA, a dot-like staining pattern was observed for LAMP2A.

Similarly, we generated HSC70 knockdown and overexpression cells. We transduced NB4 acute promyelocytic leukemia (APL) cells with a control and three independent shRNAs targeting HSC70. Only shHSC70_3 transduced NB4 cells showed a reduction in HSC70 expression compared to the control transduced cells on a western blot (Figure 2(a)). This knockdown was confirmed by immunohistochemical staining of HSC70 (Figure 2(b)). A transient overexpression of an HSC70 expression plasmid in 293 T cells resulted in increased protein expression as assessed by western blotting and immunohistochemistry (Figures 2(c) and 2(d)). Together, our knockdown and overexpression experiments in different cell lines underline the specificity of the anti-LAMP2A and anti-HSC70 antibodies used in immunohistochemical staining.

### 3.2. LAMP2A and HSC70 Expression and Intratumoral Heterogeneity

For determining LAMP2A and HSC70 expression in 336 pSQCC, a total of 1399 TMA cores stained with LAMP2A and 1378 TMA cores stained with HSC70 were available for evaluation. LAMP2A expression was absent in 109/1399 (8%) of the TMA cores, weak in 286/1399 (20%), medium in 683/1399 (49%), and strong in 321/1399 (23%) cores. The intensity of HSC70 was weak in 146/1378 (11%), medium in 510/1378 (37%), and strong in 708/1378 (51%) of the TMA cores. Only 14/1378 (1%) of the TMA cores lacked HSC70 expression. IRS multiplying intensity scores with the extent of tumor staining were calculated as described in the Material and Methods. For subsequent analysis, the IRS of the single cores was used for assessing intratumoral heterogeneity and the correlation between the two markers. For the determination of the expression levels with clinicopathologic characteristics, the IRS sum scores were calculated for each tumor. Correlations were performed using either the IRS sum scores or a categorization based on the quartiles of the IRS sum scores.

We identified only 8 cases with additional nuclear expression of HSC70 (Figures 3(i) and 3(m)), and in this small group, there was no statistically significant correlation with other pathological parameters or any valuable prognostic significance.

There was no significant intratumoral staining heterogeneity for LAMP2A and HSC70 when comparing the IRS of the single cores per tumor of the respective markers among each other (*p* values between 0.155 and 0.82). Rather, there was a highly significant correlation for the IRS within the four TMA cores per tumor for LAMP2A (*r* = range 0.751-0.895; *p* < 0.001 each) and the IRS of HSC70 (*r* = range 0.428-0.698; *p* < 0.001 each).

### 3.3. Correlation between LAMP2A and HSC70

Due to a close cooperation of LAMP2A and HSC70 on the molecular level, the IRS scores of these markers were compared. There was no significant correlation between the expression of LAMP2A and HSC70 in the single cores and overall (*p* values between 0.388 and 0.875; overall: *p* = 0.68).

### 3.4. Correlation between LAMP2A and HSC70 Expression Levels and Pathological Parameters

For the assessment of associations between LAMP2A and HSC70 expression and pathological parameters, the IRS scores of each tumor (i.e., the sum of all IRS scores across all TMA cores per tumor) were calculated against the respective factors or were subdivided into quartiles for a categorization into low (lower three quartiles) to high (highest quartile) expression levels. In UICC pT1a tumors, the least advanced subgroups of tumors in the pT category, higher LAMP2A levels and lower HSC70 levels, were observed, but this was overall not statistically significant when analyzing the entire cohort. For all other pT categories, IRS levels were within a comparable range. Similarly, there was no significant association between the expression of LAMP2A and HSC70 with other pathological parameters such as pN categories, presence of distant metastases, UICC/AJCC TNM staging and grading, nor with gender or patients' age. These results were observed using both calculation methods (IRS sum scores and categorization; see supplemental Figures S1- S10).

### 3.5. Correlation with Survival

Survival data was available for 254 patients. Mean disease free survival (DFS) was 50.1 months, and mean overall survival (OS) was 53.9 months. Survival analysis for DFS and OS was calculated using the expression levels defined by the four quartiles. The best prognostic discrimination was seen for the fourth quartile (then defined as high expression) versus the lower three quartiles (defined as low expression). This threshold was then used for further analysis. IRS cutoffs for differentiating between low and high staining were 28.0 (summarized from all four tumor cores) for LAMP2A and IRS 41.8 for HSC70. Low levels of LAMP2A staining (lower three quartiles) were observed in 255/336 (76%) cases and high levels in 81/336 (24%) cases. Similarly, we found low levels of HSC70 (lower three quartiles) in 252/336 (75%) cases and high levels of HSC70 in 84/336 (25%) cases.

**Figure 4:**
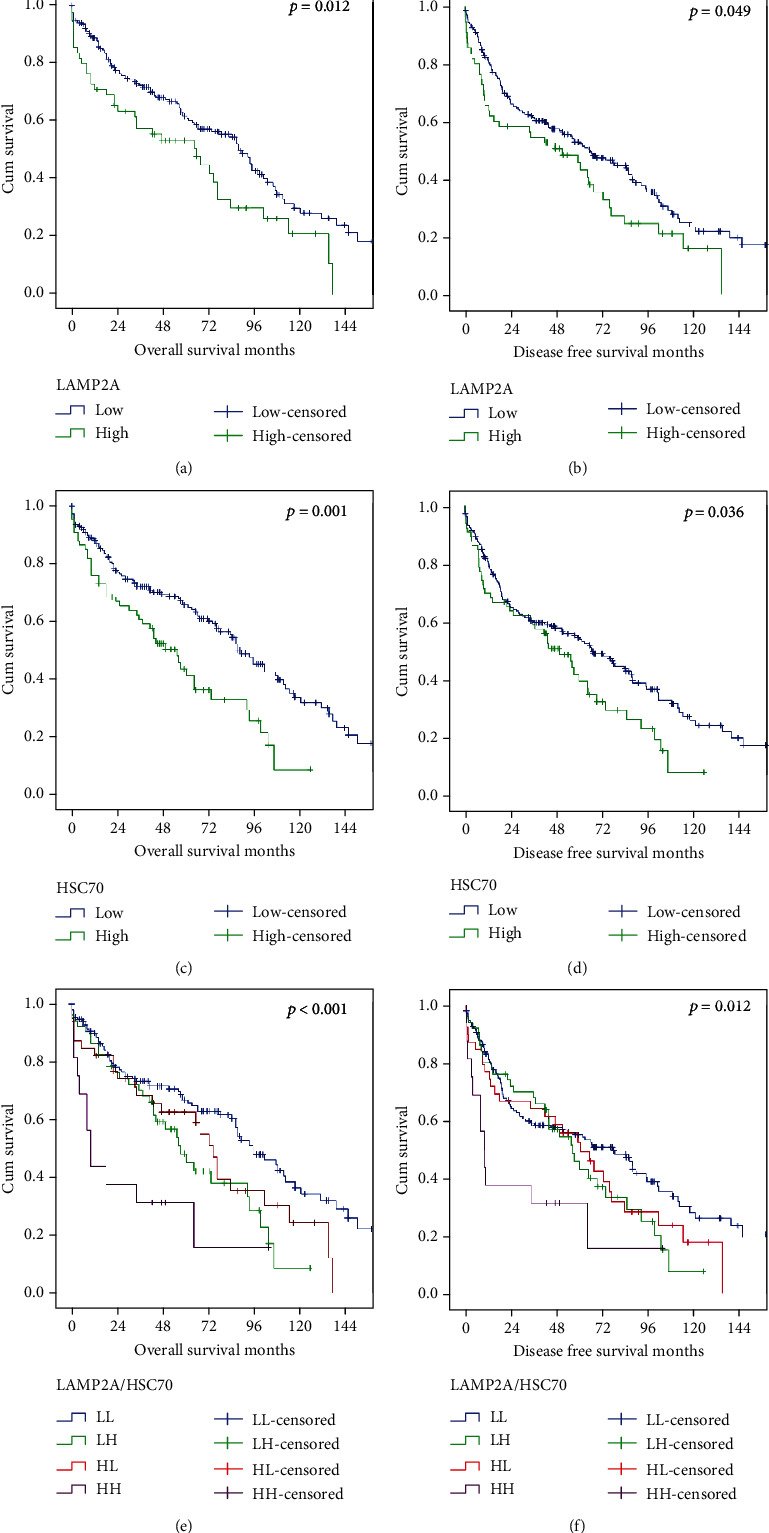
Kaplan-Meier curves (overall survival and disease free survival) for expression of autophagy-related proteins: (a) OS, LAMP2A; (b) DFS, LAMP2A; (c) OS, HSC70; (d) DFS, HSC70; (e) OS, combined; (f) DFS, combined.

High LAMP2A levels were associated with unfavorable OS (*p* = 0.012) and DFS (*p* = 0.049). High HSC70 levels were also associated with worse outcome, including OS (*p* = 0.001) and DFS (*p* = 0.036) (Figures 4(a)–4(d)).

Applying multivariate analysis, both markers were also independent adverse prognostic factors for OS and superior to UICC/AJCC TNM stage (Table 2). For DFS, both markers, but also UICC/AJCC TNM stage, were independent prognostic factors (Table 3).

The combination of LAMP2A and HSC70 showed an even more significant prognostic impact: patients with LAMP2Ahigh/HSC70high tumors showed the worst prognosis and patients with LAMP2Alow/HSC70low tumors the best prognosis (*p* < 0.001 for OS and *p* = 0.012 for DFS, Figures 4(e) and 4(f)). This combination was also an adverse independent prognostic factor for OS and DFS in multivariate analysis (Tables 4 and 5).

## 4. Discussion

The role of autophagy and its subtypes, particularly CMA, in tumorigenesis is complex. As described in previous studies, it may play a dichotomous role in cancer by suppressing the initiation of tumor growth but promoting tumor growth and survival in established cancers [33]. The expression patterns of CMA markers are in the majority still unknown but could contribute to a better understanding of these complex and fine-tuned cellular mechanisms.

In our retrospective study, we assessed the immunohistochemical expression patterns of the two CMA key players LAMP2A and HSC70 and their prognostic value in primary resected pSQCC. The strength of our study is the large and histologically homogeneous, well-curated patient cohort with survival data available for 254 cases and the meticulous validation of specificity of the immunohistochemical markers used. In order to guarantee the reliability of our results [34], we generated overexpression and knockdown cell lines for each marker and validated protein expression patterns via western blot and immunohistochemistry on FFPE cell pellets.

We could demonstrate a variable expression of LAMP2A and HSC70 in pSQCC. There was a wide spectrum of staining intensity, even though the best prognostic stratification was observed between strong positive tumors versus all other staining patterns. Our key finding is that both markers, LAMP2A and HSC70, are independent adverse prognostic markers in pSQCC including OS and DFS. The combination of both of them (LAMP2Ahigh/HSC70high) showed an even more significant prognostic impact, although this marker profile was observed in only few cases (*n* = 21). This marker profile might correspond to activated CMA in advanced tumors, which could be required to overcome the altered metabolism of the tumor cells [13].

Moreover, we found no significant intratumoral heterogeneity of LAMP2A and HSC70 staining in the examined tumors. There was no significant correlation between these two markers and other pathological parameters. Surprisingly, there was also no correlation between these two individual markers, although biologically the two proteins cooperate in CMA, which underlines the need of further functional studies in this field. It is important to mention that high expression levels of HSC70 and LAMP2A are considered indicative of high CMA levels but are not a proof of high CMA activity. As CMA is a dynamic process, it cannot be captured completely using a static method as immunohistochemical staining. High levels of CMA markers could as well occur in a situation of stalled CMA degradation for instance if the lysosomal function is impaired.

Similar results have been reported in other tumor types. LAMP2A is expressed in almost all types of tumors, but the prognostic value of tumoral expression has not been extensively explored yet. In a recent study on esophageal squamous cell carcinomas, high expression of LAMP2A was associated with poor prognosis, similar to our findings [35].

High expression levels of HSC70 were observed in many cancers, e.g., hepatocellular or colon carcinomas [36, 37]. HSC70 was described as a prognostic marker in colorectal cancer (favorable), liver cancer (unfavorable), and renal cancer (favorable) [38]. In our cohort of pSQCC, HSC70 expression was associated with an unfavorable prognosis. This discrepancy of the prognostic value of HSC70 might result from the diversity of HSC70 function in the cell [11].

In lung cancer, the expression of HSP70 was studied before, another HSP70 family member also known as HSPA1A or HSP70-1. However, the results are inconsistent, including a reported better prognosis in patients with HSP70-positive NSCLC as well as an association with a Ki-67 proliferation index and nuclear HSP70 expression [39, 40]. Yet, intense focus has been placed on exploring the potential of HSP70 inhibitors as chemotherapeutic agents [10]. For HSC70, however, pharmacological inhibitors were not available until few years ago. Since this protein is as well involved in the presentation of antigenic peptides by major histocompatibility complex class II (MHCII), it was recently exploited as a target for the treatment of autoimmune disorders [11]. In this context, a phosphopeptide called P140 was shown to directly interact with HSC70 and to inhibit CMA. This new drug showed a significant downregulation of the signaling of autoreactive T cells in vivo in a model of systemic lupus erythematosus, leading to a remarkable improvement of the pathophysiologic condition [41]. Thus, HSC70 may be a possible target to inactivate CMA in future anticancer therapy, warranting the current detailed expression analyses. If those aggressive pSQCC with high LAMP2A and HSC70 expression might be candidates for the new CMA-targeting therapeutics must be further evaluated in functional analyses and subsequent clinical studies.

Our present work has some limitations warranting subsequent validation studies. Importantly, evaluation of the stainings was performed by only one pathologist. Although this ensures the application of homogenously calibrated criteria in scoring of all cases, it precludes any statement on interobserver variability of the scoring method. This will be addressed in the subsequent studies. Additionally, there is a possible bias in the sample collection inherent in the retrospective design of the study, although all consecutive cases were included. Finally, evaluation of the stainings was performed on a TMA, which might not represent the entire tumor when compared to the whole slide. In order to minimalize this limitation, a minimum of 4 cores per tumor were evaluated separately. A comparison of scores between the different cores per tumor failed to show a significant staining heterogeneity, speaking in favor of the robustness of the staining pattern throughout the whole tumor [42, 43].

From a biological point of view, the results of our tissue-based explorative study underline the role of CMA in human tumorigenesis. From a clinical point of view, the two markers HSC70 and LAMP2A may be exploited as prognostic biomarkers in pSQCC.

## 5. Conclusions

In our present study, we demonstrated the variable immunohistochemical expression of the key CMA markers LAMP2A and HSC70 in pSQCC. High expression levels of these markers were associated with worse prognosis, including OS and DFS, and could be considered as biomarkers for potential future CMA inhibiting therapies.

## Figures and Tables

**Table 1:** Description of the case collection.

		*n*	%
Gender	m	286	85.1
	f	50	14.9

Median age (range)	69 (43-85)		

pT UICC 2017	pT1a	6	1.8
	pT1b	21	6.3
	pT1c	45	13.4
	pT2a	68	20.2
	pT2b	51	15.2
	pT3	78	23.2
	pT4	67	19.9

pN UICC 2017	pN0	192	57.1
	pN1	107	31.9
	pN2	37	11.0

Distant metastases	Absent	328	97.6
	Present	8	2.4

AJCC/UICC TNM stage 2017	IA1	4	1.2
	IA2	17	5.1
	IA3	31	9.2
	IB	48	14.3
	IIA	28	8.3
	IIB	88	26.2
	IIIA	86	25.6
	IIIB	26	7.7
	IIIC	0	0.0
	IVA	6	1.8
	IVB	2	0.6

Grading	Grade 1	7	2.1
	Grade 2	170	50.6
	Grade 3	159	47.3

Resection status	R0	292	86.9
	R1/R2	44	13.1

Total		336	100.0

**Table 2:** Results of multivariate analysis for OS.

	HR	95% CI		*p* value
		Lower	Upper	
Gender	0.578	0.337	0.990	0.046
Age	2.550	1.785	3.642	<0.001
UICC/AJCC stage 2017 (I, II, III, IV)	1.225	0.974	1.539	0.083
R status	1.705	1.062	2.736	0.027
LAMP2A^high^	2.059	1.396	3.036	<0.001
HSC70^high^	1.987	1.368	2.885	<0.001

**Table 3:** Results of multivariate analysis for DFS.

	HR	95% CI		*p* value
		Lower	Upper	
Gender	0.682	0.423	1.102	0.118
Age	2.059	1.486	2.851	<0.001
UICC/AJCC stage 2017 (I, II, III, IV)	1.233	1.001	1.520	0.049
R status	1.591	1.021	2.480	0.040
LAMP2A^high^	1.709	1.185	2.467	0.004
HSC70^high^	1.484	1.046	2.105	0.027

**Table 4:** Results of multivariate analysis for OS and LAMP2A/HSC70 marker combination.

	HR	95% CI		*p* value
		Lower	Upper	
Gender	0.581	0.338	0.997	0.049
Age	2.629	1.843	3.751	<0.001
UICC/AJCC stage 2017 (I, II, III, IV)	1.221	0.971	1.536	0.088
R status	1.764	1.100	2.828	0.018
LAMP2A^high^/HSC70^high^	1.529	1.287	1.816	<0.001

**Table 5:** Results of multivariate analysis for DFS and LAMP2A/HSC70 marker combination.

	HR	95% CI		*p* value
		Lower	Upper	
Gender	0.688	0.426	1.110	0.125
Age	2.085	1.508	2.882	<0.001
UICC/AJCC stage 2017 (I, II, III, IV)	1.231	0.998	1.517	0.052
R status	1.612	1.035	2.509	0.035
LAMP2A^high^/HSC70^high^	1.342	1.140	1.579	<0.001

## Data Availability

The primary data used to support the findings of this study are available from the corresponding author upon request.
