# Corrigendum to “Chaperone-Mediated Autophagy Markers LAMP2A and HSC70 Are Independent Adverse Prognostic Markers in Primary Resected Squamous Cell Carcinomas of the Lung”

**DOI:** 10.1155/2021/9846486

**Published:** 2021-11-30

**Authors:** Tereza Losmanová, Félice A. Janser, Magali Humbert, Igor Tokarchuk, Anna M. Schläfli, Christina Neppl, Ralph A. Schmid, Mario P. Tschan, Rupert Langer, Sabina Berezowska

**Affiliations:** ^1^Institute of Pathology, University of Bern, Bern 3008, Switzerland; ^2^Graduate School for Cellular and Biomedical Sciences, Bern 3012, Switzerland; ^3^Division of General Thoracic Surgery, Inselspital University Hospital Bern, Bern 3010, Switzerland; ^4^Department of Biomedical Research (DBMR), University of Bern, Bern 3008, Switzerland; ^5^Institute of Pathology and Molecular Pathology, Kepler University Hospital, Johannes Kepler University Linz, 4021 Linz, 4040 Linz, Austria; ^6^Institut de Pathologie, Centre HospitalierUniversitaire Vaudois et Université de Lausanne, Lausanne 1011, Switzerland

In the article titled “Chaperone-Mediated Autophagy Markers LAMP2A and HSC70 Are Independent Adverse Prognostic Markers in Primary Resected Squamous Cell Carcinomas of the Lung” [1], the authors identified an error in the name of the manufacturer of the antibody mentioned in Materials and Methods. The following should be corrected from

“LAMP2A (Novus Biologicals, Zug, Switzerland, rabbit polyclonal, #NB600-1384)

HSC70 (LabForcembl, Nunningen, Switzerland, rabbit polyclonal, #PM0045)”

to

“LAMP2A (Abcam, Cambridge, UK, rabbit polyclonal, ab18528)

HSC70 (Thermo Fisher Scientific, Waltham, MA, USA, mouse monoclonal, MA3-014)”
