# Supplementary material for: Chaperone-Mediated Autophagy Markers LAMP2A and HSC70 Are Independent Adverse Prognostic Markers in Primary Resected Squamous Cell Carcinomas of the Lung
Source: Oxid Med Cell Longev. 2020 Sep 21;2020:8506572. doi: 10.1155/2020/8506572 (PMC7527932; doi:10.1155/2020/8506572)
Supplement: Supplementary Materials — The following are available online, Figures S1-S10: IRS values of LAMP2A and HSC70 with pathological parameters (TNM categories, Grading, Stage). [file 8506572.f1.docx]

Supplemental Data File


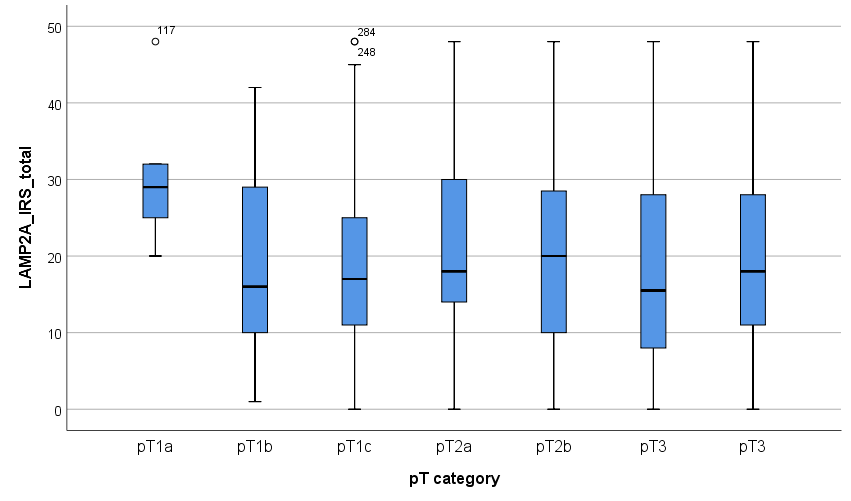


**Figure S1.** IRS values of LAMP2A and pT category


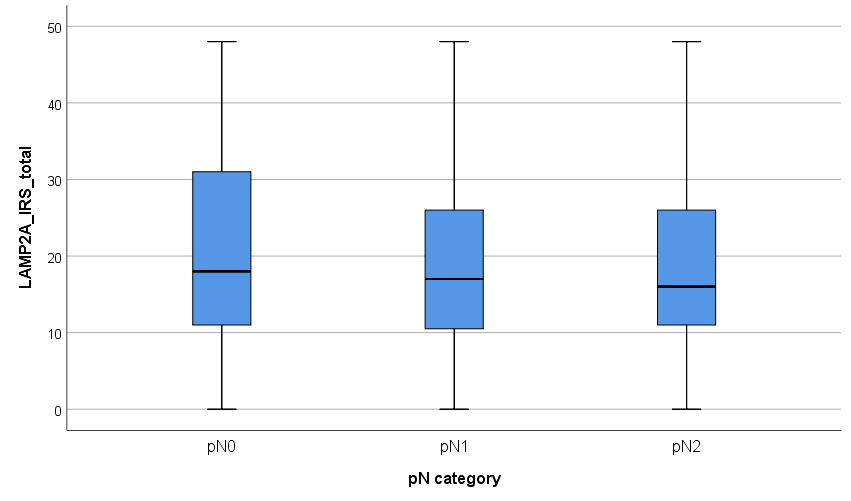


**Figure S2.** IRS values of LAMP2A and pN category


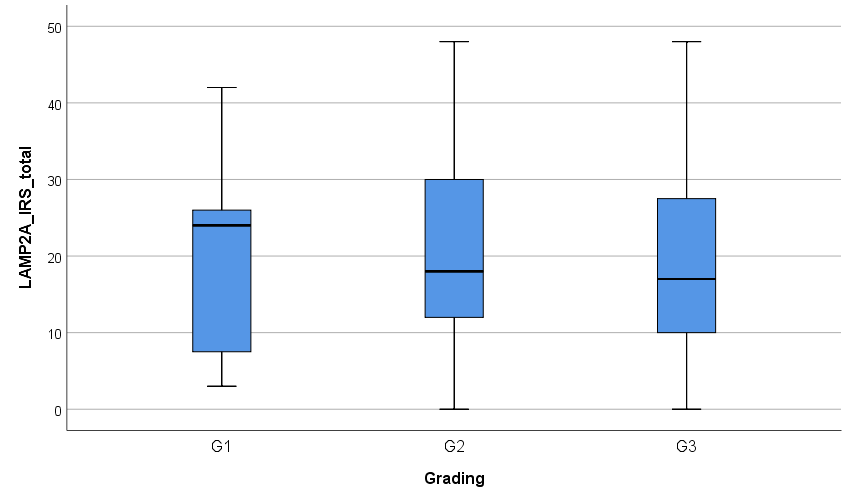


**Figure S3.** IRS values of LAMP2A and Grading


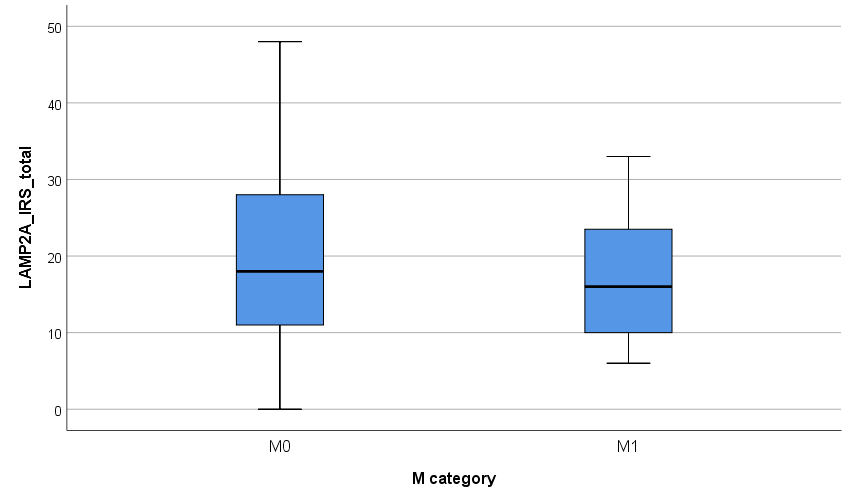


**Figure S4.** IRS values of LAMP2A and M category


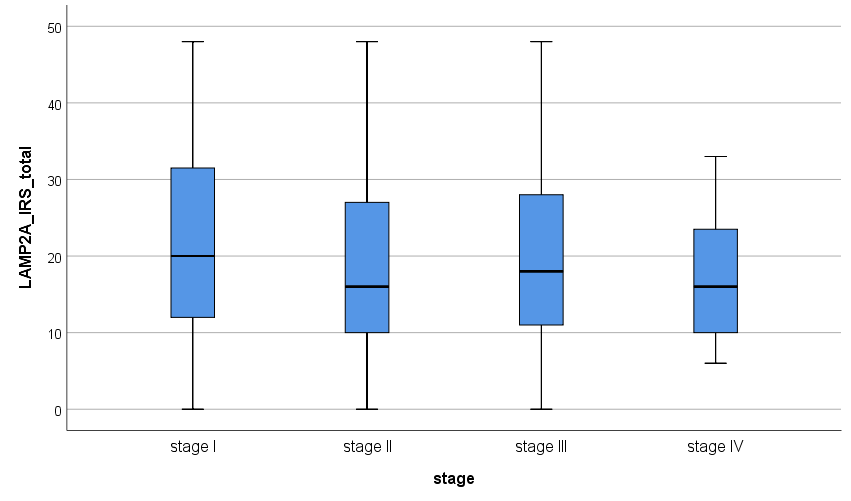


**Figure S5.** IRS values of LAMP2A and UICC/AJCC Stage 2017 (I, II, III, IV)


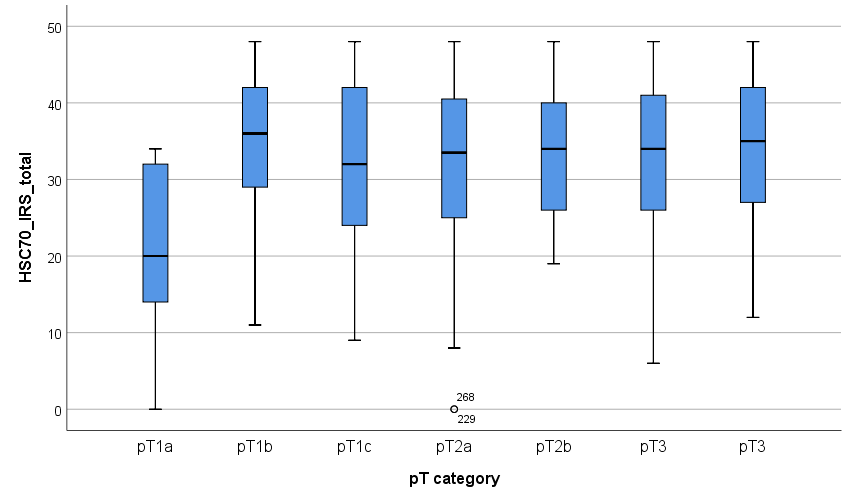


**Figure S6.** IRS values of HSC70 and pT category


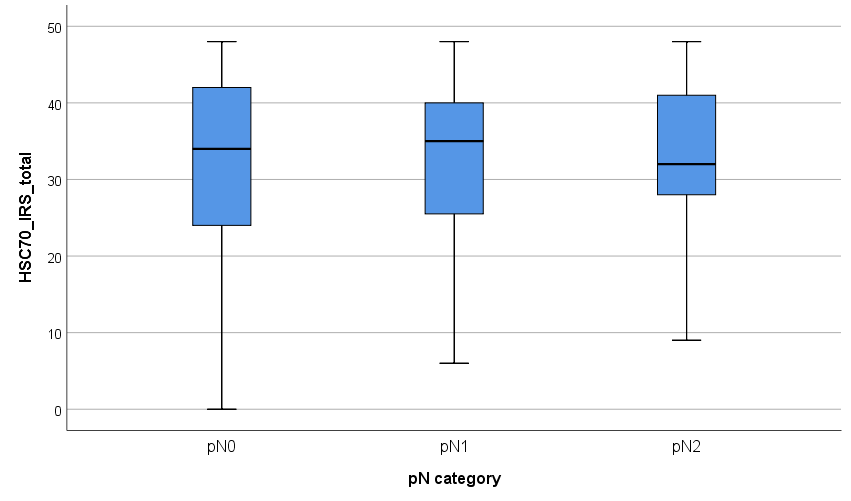


**Figure S7.** IRS values of HSC70 and pN category


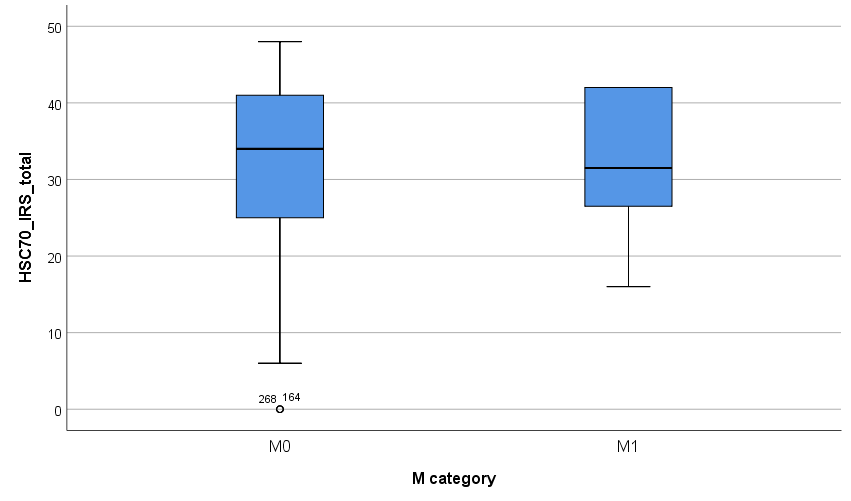


**Figure S8.** IRS values of HSC70 and M category


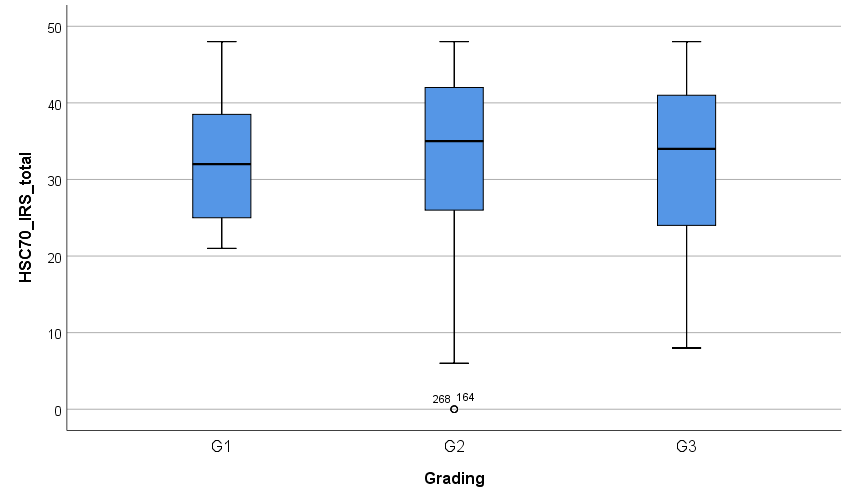


**Figure S9.** IRS values of HSC70 and Grading


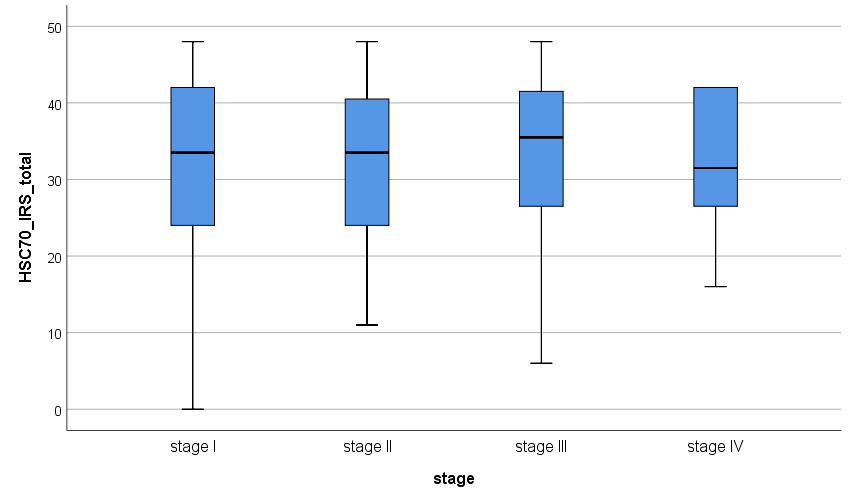


**Figure S10.** IRS values of HSC70 and UICC/AJCC Stage 2017 (I, II, III, IV)
